# Hemiplegia in acute ischemic stroke: A comprehensive review of case studies and the role of intravenous thrombolysis and mechanical thrombectomy

**DOI:** 10.1002/ibra.12146

**Published:** 2024-01-13

**Authors:** Karen Adriana Carrillo Navarrete, Christian Chapa González

**Affiliations:** ^1^ Instituto de Ingeniería y Tecnología Universidad Autónoma de Ciudad Juárez Ciudad Juárez, Chihuahua México; ^2^ Grupo de Nanomedicina, Laboratorio de Integración de Datos y Evidencia en Revisiones de Salud y Ciencia, LIDERSC Universidad Autónoma de Ciudad Juárez Ciudad Juárez, Chihuahua México

**Keywords:** brain, hemiplegia, ischemic stroke, thrombectomy, thrombolytic therapy

## Abstract

Acute ischemic stroke is a significant health concern worldwide, often leading to long‐term disability and decreased quality of life. Rapid and appropriate treatment is crucial for achieving optimal outcomes in these patients. Intravenous thrombolysis (IVT) and mechanical thrombectomy (MT) are two commonly used interventions for acute ischemic stroke, but their effectiveness in improving neurological symptoms and functional outcomes in patients with hemiplegia remains uncertain. The aim of this work was to evaluate the impact of IVT and MT within a 4.5‐h time frame on patients with acute ischemic stroke and hemiplegia. A systematic review was conducted following the Preferred Reporting Items for Systematic Reviews and Meta‐Analyses guidelines. Relevant studies that assessed the impact of IVT and MT within 4.5‐h on hemiplegia in patients with an acute ischemic stroke were included. Data were extracted and analyzed to determine the overall effects of these interventions. Most included case reports indicate positive outcomes in terms of neurological symptom improvement and functional recovery in patients with hemiplegia after receiving IVT and MT within the specified time frame. However, the heterogeneity among the patients and the limited use of IVT due to contraindications posed challenges in determining the most effective treatment option. The findings from the included studies demonstrate that both interventions led to a decrease in National Institutes of Health Stroke Scale scores, indicating an improvement in neurological symptoms. The results highlight the beneficial effects of early thrombolytic interventions and MT on the neurological status and functional outcomes of patients with an acute ischemic stroke.

## Graphical abstract

The accompanying paper presents a comprehensive review of case studies focusing on the impact of intravenous thrombolysis and mechanical thrombectomy within a 4.5‐h time frame on hemiplegia in patients with acute ischemic strokes. The study evaluates the effectiveness of these interventions in improving neurological symptoms and functional outcomes. It also discusses the challenges and considerations in selecting the most appropriate treatment for patients with hemiplegia, providing valuable insights for stroke management. The graphical abstract visually represents the key aspects of the study, showcasing the ischemic stroke, thrombolysis, thrombectomy, National Institutes of Health Stroke Scale, and the significant association of hemiplegia with acute ischemic stroke.

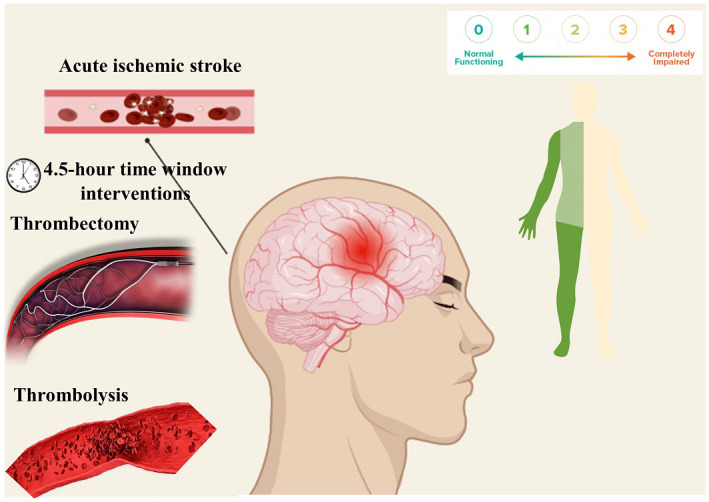

## 1 INTRODUCTION

Ischemic strokes are one of the most prevalent cerebrovascular conditions, significantly contributing to global mortality and disability.^1^, ^2^ Ischemic strokes encompass a cascade of pathological events, encompassing excessive calcium accumulation,^3^ excitotoxicity,^4^ increased oxidative stress,^5^, ^6^ mitochondrial dysfunction,^7^ inflammation,^8^, ^9^, ^10^ and cell death.^11^ Acute ischemic stroke is a severe medical condition characterized by the interruption of blood flow to the brain due to arterial obstruction.^12^ Timeliness is decisive in stroke management, where early treatment within the first hours of symptoms is pivotal. This window offers an opportunity for reperfusion therapies to restore blood flow and limit cerebral damage.^13^ Currently, the two primary therapies employed during this stage are intravenous thrombolysis (IVT)^14^, ^15^ and mechanical thrombectomy (MT).^16^, ^17^, ^18^, ^19^

IVT involves the administration of a thrombolytic agent, such as tissue plasminogen activator (tPA), which dissolves the clot obstructing the cerebral artery. IVT has been demonstrated to improve outcomes in patients with acute ischemic stroke within the first 4.5‐h.^14^, ^15^, ^20^, ^21^, ^22^ In 1996, the Food and Drug Administration (FDA) approved intravenous‐tPA (IV‐tPA) for treating acute ischemic stroke, based on successful outcomes in the National Institute of Neurological Disorders and Stroke study.^23^, ^24^ Recently, MT, a type of endovascular treatment for stroke, has gained prominence as a remarkably effective therapy.^25^ MT is a procedure in which a catheter is utilized to physically extract the clot obstructing the cerebral artery. It is considered an appropriate therapeutic option within the first 24 h of symptom onset,^26^, ^27^ and some studies have suggested that it has been regarded as an adequate clinical option within the 4.5‐h time window in select patients.^28^ In the available literature, case reports have documented the utilization of MT as an effective intervention for promoting recovery in patients afflicted with acute ischemic stroke accompanied by unilateral paralysis (hemiplegia)^29^ and using both IVT and MT at the same time.^30^

Hemiplegia significantly impairs motor function and quality of life. Acute ischemic stroke impact on hemiplegia is linked to brain hemisphere lesions and peripheral vasoreactivity.^31^ These lesions can alter vasoreactivity, causing peripheral edema. Early IVT or MT significantly influences the prognosis of hemiplegic patients, which is determinant for their recovery. Although there are instruments such as the modified Rankin Scale (mRS)^32^ and the Glasgow Coma Scale^33^, ^34^ (GCS), the National Institutes of Health Stroke Scale^35^ (NIHSS) is an effective and reliable tool for assessing stroke severity, treatment, and prognosis through a simple and rapid clinical evaluation.

Acute ischemic stroke has been the focus of research on detection,^36^, ^37^ effective treatment strategies,^38^ and improved recovery.^39^, ^40^ Endovascular therapy improves outcomes and recanalizes cerebral arteries; yet, its superiority over standard intravenous tPA for disability‐free survival at 3 months is debated.^41^ Moreover, the study of Fischer et al.^42^ found that thrombectomy alone was not as effective as the combination of intravenous alteplase plus thrombectomy in achieving successful reperfusion and improving outcomes in patients with acute ischemic stroke.

Several reviews have been conducted recently in the field of stroke management,^43^ focusing on topics such as early recognition,^44^, ^45^, ^46^, ^47^, ^48^ novel therapeutics,^49^, ^50^, ^51^ comparison of thrombolytic agents,^52^ combination of MT with IVT,^53^ and safety and efficacy of IVT after antagonization of unfractionated heparin with protamine,^54^ among others. This review critically assesses IVT versus MT within a 4.5‐h window, considering pretreatment and posttreatment NIHSS scales—an aspect overlooked in current literature. This systematic review aims to answer the next core question: What therapy administered within the first 4.5‐h after the onset of acute ischemic stroke yields superior outcomes in terms of prognosis for patients with hemiplegia, as measured by functional recovery and quality of life?

## 2 METHODS

### 2.1 Search strategy

This systematic review was conducted following the Preferred Reporting Items for Systematic Reviews and Meta‐analysis (PRISMA) statement, and it was registered at PROSPERO (registration number: CRD42023436985). A systematic search for relevant records was conducted using multiple reputable databases, including ScienceDirect, EBSCOhost, Nature, PubMed, and Ovid. These databases were selected for their comprehensive coverage of scientific literature. By utilizing these databases, a search strategy was employed to gather pertinent records for the review of the topic at hand. The following search strategy was defined (“Acute ischemic stroke”) AND (treatment OR therapy) AND (hemiplegia OR hemiplegic) AND (“symptom onset” OR “therapeutic window”). Different automated filters were applied to the search results based on the respective databases, considering only records published from 2012 to 2022. The review included research articles, case reports, trials, and clinical studies.

### 2.2 Eligibility assessment

In the systematic review, specific inclusion and exclusion criteria were defined to narrow down the search and compilation of records from different databases, aiming to obtain more specific information to answer the research question. The review included records with cases of patients aged between 18 and 65 years, who experienced an acute ischemic stroke and received treatments or therapies within the first 4.5‐h after symptom onset. Records were considered if they included patients with hemiplegia and if neurological deficit and functional outcome after the stroke were measured using scales such as GCS, NIHSS, and mRS. Exclusion criteria involved records with patients having hemorrhagic strokes, those providing preventive measures for stroke, and those without full access.

### 2.3 Data extraction

The collected records underwent a full data analysis. Relevant information from these records was organized in an Excel table, including details such as the publication year, authors, neurological deficit scores measured using scales before treatment, specific types of neurological deficits observed in patients, the therapy or treatment administered for acute ischemic stroke, the therapeutic window within which it was administered (less than 4.5‐h), and the neurological deficit scores following the therapy. Upon reviewing the selected records obtained during the search, relevant data were extracted for analysis. This included information on the authors, year of publication (encompassing records from 2012 to 2022), reference sources, applied therapy or treatment, therapeutic window or time from symptom onset (restricted to less than 4.5‐h), neurological deficit observed, neurological deficit score before the therapy, and neurological deficit score after the therapy.

The selection and screening of records were carried out by two independent reviewers. The resulting records were downloaded in Research Information Systems (RIS) format and imported into Mendeley, where duplicates were identified and removed. Subsequently, the information from the records was exported to Microsoft Excel for further screening. During this stage, each record's abstract was carefully examined by two reviewers, following predefined inclusion and exclusion criteria outlined in the protocol. In cases where there were discrepancies in data extraction or uncertainties, discussions with colleagues were undertaken to achieve consensus.

### 2.4 Quality assessment

To evaluate the methodological quality and risk of bias in the included case reports, the Joanna Briggs Institute (JBI) Critical Appraisal Checklist for Case Reports was utilized. This comprehensive checklist comprises eight specific questions designed to assess various aspects of the case reports' methodology and reporting. Each question was carefully addressed by examining the information provided in the selected records. The JBI checklist enabled a systematic and rigorous evaluation of the methodological quality of the case reports, ensuring a thorough assessment of potential biases and enhancing the overall reliability of the findings.

## 3 RESULTS

### 3.1 Study identification

**Figure 1:**
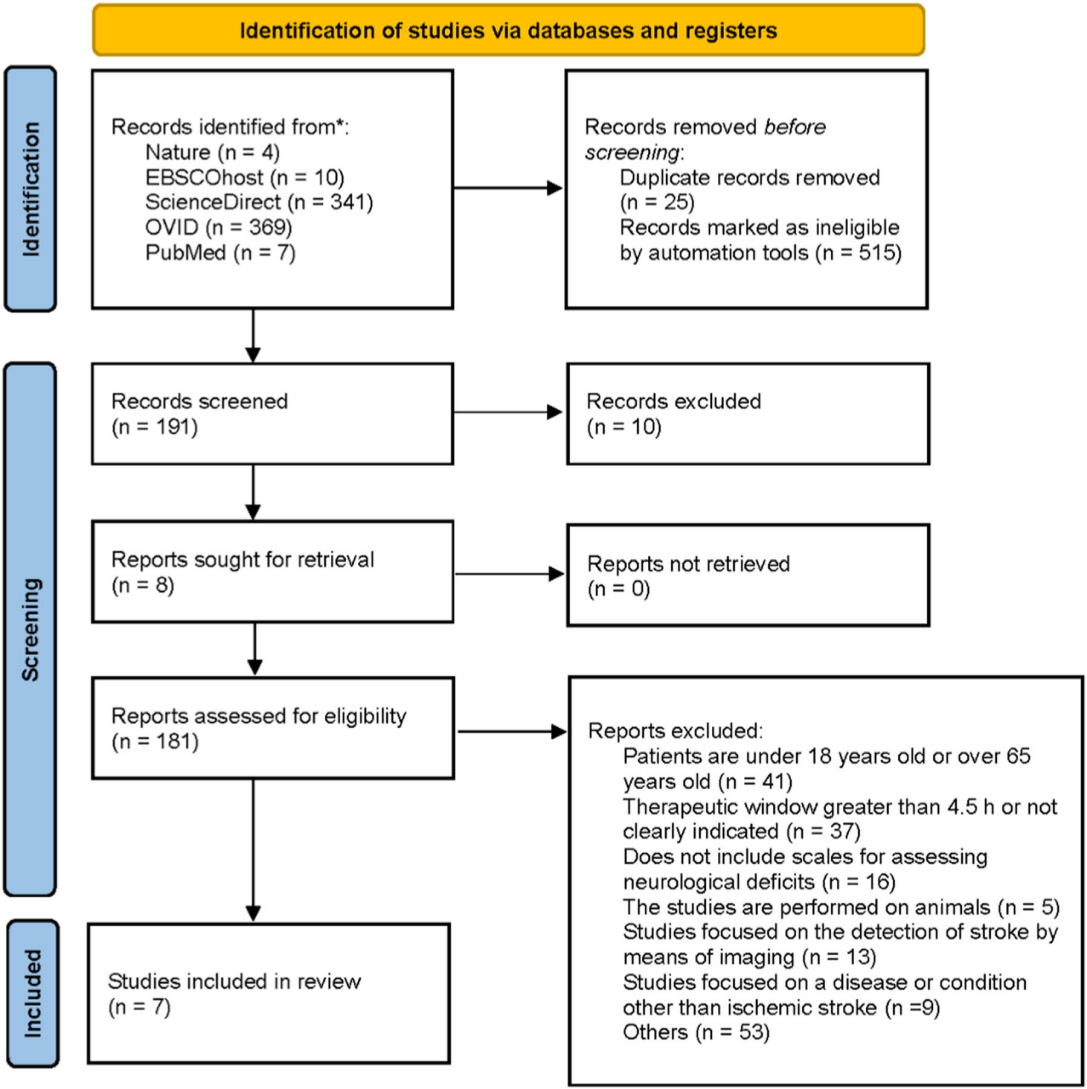
A flowchart diagram, adhering to the PRISMA guidelines, illustrates the number of studies that underwent filtering, starting from the identification stage and culminating in their final inclusion in the analysis.

To ensure robust scientific rigor in the results section of this study, an extensive search was conducted across multiple databases, including ScienceDirect, PubMed, Ovid, EBSCOhost, and Nature. The search strategy employed Boolean operators to refine the retrieval of relevant records. Figure 1 illustrates the varying numbers of records obtained from each database. Applying automated filters, we refined the results based on the publication year (2012 to 2022), record type, and, when applicable, patient age. Out of the initial pool of records, 515 were excluded as they did not meet the predefined criteria, and 25 duplicate records were removed. Subsequently, a total of 191 records underwent a meticulous screening process, with 10 identified as systematic reviews. Interlibrary loan requests were made for eight records, successfully acquiring them for further analysis. The remaining 181 records underwent thorough evaluation to determine their eligibility for inclusion. After the thorough evaluation, a total of seven studies were identified that met all the predefined criteria and were included in the analysis.^55^, ^56^, ^57^, ^58^, ^59^, ^60^, ^61^ These studies satisfied the inclusion criteria regarding patient characteristics, therapeutic window, neurological deficit assessment, and treatment approaches.

### 3.2 Characteristics of individual studies

This section presents an exhaustive analysis of each of the studies, highlighting their characteristics and unique contributions, presented in chronological order. The eligible records included in this study span from 2013 to 2022, as evident in Table 1, which presents the concise summaries of the studies and is organized according to the year of publication. These records include clinical cases of patients with acute ischemic stroke aged 24–62 years, with their neurological deficits assessed using the NIHSS (Table 2). It is noteworthy that the included records specifically focus on patients presenting with hemiplegia as the primary symptom and exhibit a time interval of less than 4.5‐h between symptom onset and treatment initiation. The majority of patients received IVT and/or MT as therapeutic interventions. Furthermore, these records provided assessments of patient prognosis using the aforementioned scale, even extending to follow‐ups conducted over days or months. It is important to mention that out of the seven included records in this review, only references^56^ and^60^ explicitly mention obtaining informed consent from the patients.

**Table 1:** Case descriptions of unique interventions in acute ischemic stroke. [Correction added on 2 March 2024, after first online publication: The table 1 has been revised in this version.]

References	Study description
Vasko, P. et al. (2013)^55^	A 46‐year‐old woman with hormonal contraception experienced severe acute hemispheral stroke. Prompt percutaneous intervention led to full recovery.
Sukockienė, E. et al. (2016)^56^	Aortic dissection can present as acute cerebral infarction. Carotid ultrasound played a key role in diagnosing aortic dissection in a follow‐up case.
Aaron, S. et al. (2016)^57^	Two pregnant patients with acute ischemic stroke were successfully treated with mechanical clot retrieval using the Penumbra system.
Ohya, Y. et al. (2018)^58^	The use of idarucizumab prior to thrombolytic therapy showed improved neurological symptoms in a patient with cardioembolic stroke under dabigatran.
Balci, S. et al. (2019)^59^	Transbrachial access was successfully used for mechanical thrombectomy in two patients with floating aortic arch thrombi and acute ischemic stroke.
Benichi, S. et al. (2019)^60^	Mechanical thrombectomy was performed through direct cervical access in a patient with limited vascular access, resulting in successful recanalization.
Sari, P. et al. (2022)^61^	Intravenous thrombolysis was safely and effectively administered to a patient with acute ischemic stroke and concomitant vertebrobasilar dolichoectasia.

**Table 2:** Hemiplegia and time from symptom onset to treatment initiation in different interventions for acute ischemic stroke. [Correction added on 2 March 2024, after first online publication: The table 2 has been revised in this version.]

References	Age	Intervention	Time from symptom onset to treatment initiation, hours	Hemiplegia
Aaron, S. et al. (2016) A.^57^	28	MT	1:20	Left side
Balci, S. et al. (2019) A.^59^	57		2:00	Right side
Benichi, S. et al. (2019)^60^	60		2:00	Right side
Balci, S. et al. (2019) B.^59^	60		1:30	Left side
Aaron, S. et al. (2016) B.^57^	24		1:00	Left side
Vasko, P. et al. (2013)^55^	46		1:22	Left side
Sarii, P. et al. (2022)^61^	62	IVT	2:45	Left side
Sukockienė E. et al. (2016)^56^	45		2:30	Left side
Ohya, Y. et al. (2018)^58^	57		1:18	Right side

Abbreviations: IVT, intravenous thrombolysis; MT, mechanical thrombectomy.

Vasko et al.^55^ reported a case of a 46‐year‐old woman with hormonal contraception who suffered a severe acute hemispheral stroke due to occlusion of the medial cerebral artery. Prompt percutaneous intervention using a stent‐retriever resulted in complete thrombectomy and subsequent full recovery within hours, without the use of thrombolysis or general anesthesia. Sukockienė et al.^56^ focused on the association between aortic dissection and acute cerebral infarction, emphasizing the importance of rapid and accurate diagnosis to enable timely thrombolytic treatment within the 4.5‐h window. The authors presented a follow‐up case where carotid ultrasound examination played a key role in diagnosing Stanford type A aortic dissection, initially presenting as an acute ischemic stroke. Using contrast‐enhanced computed tomography and transthoracic echocardiography helped monitor disease progression and guide treatment decisions.

Sanjith et al.^57^ addressed the management of acute ischemic stroke in pregnancy, highlighting the scarcity of literature on mechanical clot retrieval using the Penumbra system. The authors described two successful cases of pregnant patients with embolic stroke treated with mechanical clot retrieval, emphasizing the unique challenges and potential treatment options in this specific population. Ohya et al.^58^ investigated the use of idarucizumab, an antidote for dabigatran, before thrombolytic therapy in patients with stroke under dabigatran treatment. They presented a case of a patient with severe cardioembolic stroke treated with recombinant tissue plasminogen activator (rt‐PA) following the reversal of dabigatran with idarucizumab. Thrombolytic therapy significantly improved the patient's neurological symptoms without hemorrhagic complications, providing insights into the potential effectiveness and safety of this approach.

Balci et al.^59^ reported two cases of acute occlusion of the middle cerebral artery treated successfully with MT via transbrachial access. These patients presented unique challenges due to the presence of floating aortic arch thrombi, making transfemoral access risky. The authors emphasized the significance of assessing the aortic arch in patients with acute stroke, especially those with potential hypercoagulable states, and introduced the modified transbrachial approach as a viable alternative. Benichi et al.^60^ focused on the planning and execution of MT in patients with challenging vascular anatomy. They presented a case of a patient with acute ischemic stroke and limited access, successfully treated through direct cervical access, demonstrating the feasibility of this technique in overcoming access limitations. Sari et al.^61^ examined the impact of vertebrobasilar dolichoectasia (VBD) on IVT therapy in patients with acute stroke. They described a case involving a patient with a history of antiplatelet use and concomitant VBD who received prompt IVT, leading to a substantial improvement in neurological symptoms.

### 3.3 Synthesis of the results of included studies

All the aforementioned records mention the therapies or treatments used, which primarily include IVT and MT using stents. Only in Sukockienė et al.,^56^ a treatment aimed at stabilizing the patient's hemodynamics was utilized, proving to be beneficial. Despite IVT being the most recommended treatment for stroke within the first 4.5‐h of symptom onset, it was only used in Ohya et al.^58^ and Sari et al.^61^ It reported that stents can be employed.^56^, ^57^, ^59^, ^60^ Besides, MT can also be combined with antithrombotic treatment.^56^ IVT was contraindicated in most patients, making MT a more suitable option for the management of acute ischemic stroke.

**Figure 2:**
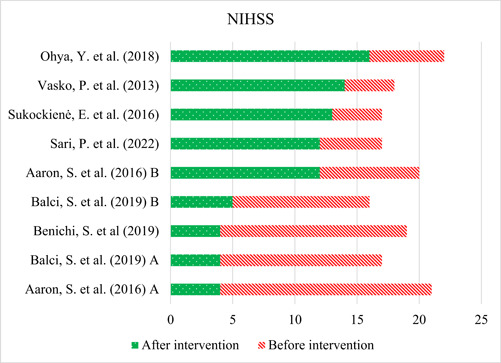
Comparison of National Institutes of Health Stroke Scale (NIHSS) scores before and after treatment in included studies. The bars in the figure represent the mean NIHSS scores before and after treatment for each study. The length of the bars reflects the magnitude of the scores. A decrease in the NIHSS score after treatment indicates an improvement in stroke severity. [Correction added on 2 March 2024, after first online publication: The figure 2 has been revised in this version.]

The included studies demonstrated the effectiveness of interventions in reducing stroke severity among the included patients. It is noteworthy that all the studies consistently reported a decrease in severity scores after the intervention. Figure 2 illustrates the NIHSS scores before and after treatment for the included studies. The NIHSS scores represented the severity of stroke, with higher scores indicating more severe strokes. The studies were sorted based on the achievement of lower NIHSS scores after treatment to better appreciate the effects of interventions.

## 4 DISCUSSION

An analysis was conducted on seven records containing case reports of individuals with acute ischemic stroke who presented with hemiplegia as a symptom and received treatment within 4.5‐h of symptom onset, primarily through MT and thrombolysis. Thrombolysis with rtPA is the most recommended treatment to be administered within a limited time after stroke symptom onset (generally within the first 4.5‐h). Thrombolysis is an effective technique in the treatment of acute ischemic stroke but can also increase the risk of intracranial hemorrhage. In the analyzed records, this therapy was used in three cases: 56, 58, and 61.^58^, ^61^ Thrombolytic treatment significantly improved the neurological symptoms of the patients without hemorrhagic complications, resulting in a decrease of 6 and 5 points, respectively, in the NIHSS score. In a case, endovascular popliteal thrombectomy was necessary on Day 4 of stroke onset due to the identification of arterial occlusion in the lower right extremity, the timing of which was unknown.^58^ In a reference, stenosis in the M1 and M2 regions of the right middle cerebral artery was observed 1 h after thrombolysis administration.^61^ In reference numbered 57, 59, and 60, MT was performed using stents, in which patients were contraindicated for thrombolysis for various reasons.^57^, ^59^, ^60^ For example, in literature 57, the patients were pregnant, and thrombolysis was contraindicated to avoid potential complications such as abortion.^57^ Patients who were not candidates for thrombolysis benefited from MT, resulting in a lower NIHSS score compared to their admission score and improvement in neurological symptoms. In literature 56, antithrombotic treatment was used during MT, resulting in a decrease of 4 points in NIHSS score, and complete recovery was shown 1 month later. Besides, other treatments, namely atropine and crystalloid fluid infusion to stabilize the patient's hemodynamic status, were also used in this study.^56^ Following these treatments, the NIHSS score decreased from 17 to 13, and the neurological deficit recovered to the state of hemiparesis. The treatments proved to be beneficial in the neurological status of the patients, resulting in a decrease in the scores of various scales without posttreatment hemorrhages. Additionally, functional capacity of the patients improved, with MT being a more viable option for these patients.

A summary of our risk of bias (RoB) assessments for the included studies is presented in Table 3. Two reviewers (KACN and CCG) independently evaluated the studies, and a good level of agreement was achieved in terms of data extraction and risk of bias analysis. Overall, the risk of bias was determined to be low in all studies.

**Table 3:** Risk of bias assessment using Joanna Briggs Institute (JBI) critical appraisal checklist for case reports. [Correction added on 2 March 2024, after first online publication: The table 3 has been revised in this version.]

Reference	1	2	3	4	5	6	7	8
Vasko, P. et al. (2013)^55^	Yes	Yes	Yes	Yes	Yes	Yes	Low	Yes
Sukockienė, E. et al. (2016)^56^	Yes	Yes	Yes	Yes	Yes	Yes	Yes	Yes
Aaron, S. et al. (2016)^57^	Yes	Yes	Yes	Yes	Yes	Yes	Low	Yes
Ohya, Y. et al. (2018)^58^	Yes	Yes	Yes	Yes	Yes	Yes	Yes	Yes
Balci, S. et al. (2019)^59^	Yes	Yes	Yes	Yes	Yes	Yes	Yes	Yes
Benichi, S. et al. (2019)^60^	Yes	Yes	Yes	Yes	Yes	Low	Yes	Yes
Sari, P. et al. (2022)^61^	Yes	Yes	Yes	Yes	Yes	Yes	Yes	Yes

In conclusion, this systematic review focused on the impact of IVT and MT within a 4.5‐h time frame on patients with acute ischemic stroke and hemiplegia. The findings highlight the positive outcomes of both interventions in improving neurological symptoms and functional outcomes in patients with acute ischemic stroke. While the majority of patients received MT, the limited use of IVT due to contraindications emphasizes the need for careful patient selection and personalized treatment strategies. The heterogeneity of the included patients and the lack of direct comparisons between therapies make it challenging to determine the most effective treatment for patients with hemiplegia. While our systematic review provides valuable insights into interventions for acute ischemic stroke with hemiplegia, the heterogeneous patient profiles and limited direct comparisons among therapies impede a definitive answer to our initial research question on the superior outcome within the first 4.5‐h.

It is important to note that the findings of this review should be interpreted in light of several limitations. The clinical heterogeneity among the included patients made it challenging to draw definitive conclusions regarding the effectiveness of specific therapies. Additionally, the limited use of IVT and the absence of direct comparisons between interventions restrict the generalizability of the results. Furthermore, the analysis relied on a small number of case reports, which may limit the overall strength of the evidence. Despite these limitations, this systematic review provides valuable insights into the therapeutic interventions for acute ischemic stroke and their impact on patients with hemiplegia.

## AUTHOR CONTRIBUTIONS

Karen Adriana Carrillo Navarrete contributed to the conceptualization, methodology, formal analysis, investigation, resources, data curation, and visualization; Christian Chapa González played a role in conceptualization, methodology, validation, formal analysis, investigation, resources, data curation, writing—original draft, writing—review and editing, supervision, and project administration.

## CONFLICT OF INTEREST STATEMENT

The authors declare no conflict of interest.

## ETHICS STATEMENT

Not applicable.

## Data Availability

The data reported in this study are available from the lead contact on request.
